# Correction: Enhanced thermal conductance at the graphene–water interface based on functionalized alkane chains

**DOI:** 10.1039/c9ra90047k

**Published:** 2019-06-17

**Authors:** Shuyu Chen, Ming Yang, Bin Liu, Min Xu, Teng Zhang, Bilin Zhuang, Ding Ding, Xiulan Huai, Hang Zhang

**Affiliations:** Institute of Engineering Thermophysics, Chinese Academy of Sciences Beijing 100190 China zhanghang@iet.cn; University of Chinese Academy of Sciences Beijing 100049 China; Materials Science, Schrödinger New York NY 10035 USA zhteg4@gmail.com; Institute of High Performance Computing, A*STAR Research Entities Singapore 138632; Institute of Materials Research and Engineering, A*STAR Research Entities Singapore 138634 ding_ding@imre.a-star.edu.sg

## Abstract

Correction for ‘Enhanced thermal conductance at the graphene–water interface based on functionalized alkane chains’ by Shuyu Chen *et al.*, *RSC Adv.*, 2019, **9**, 4563–4570.

The authors regret the omission of a funding acknowledgement in the original article. This acknowledgement is given below.

Ding Ding would like to acknowledge support from the Agency for Science, Technology and Research (A*STAR) AME Young Independent Research Grant, project: A1884c0020.

The Royal Society of Chemistry apologises for these errors and any consequent inconvenience to authors and readers.

## Supplementary Material

